# Isolated long thoracic nerve paralysis - a rare complication of anterior spinal surgery: a case report

**DOI:** 10.4076/1752-1947-3-7366

**Published:** 2009-06-23

**Authors:** Ebrahim Ameri, Hamid Behtash, Farzad Omidi-Kashani

**Affiliations:** 1 Department of Spine Surgery, Shafa Yahyaeean Hospital, Tehran, Iran; 2 Department of Orthopedic Surgery, Qaem Hospital, Mashhad, Iran

## Abstract

**Introduction:**

Isolated long thoracic nerve injury causes paralysis of the serratus anterior muscle. Patients with serratus anterior palsy may present with periscapular pain, weakness, limitation of shoulder elevation and scapular winging.

**Case presentation:**

We present the case of a 23-year-old woman who sustained isolated long thoracic nerve palsy during anterior spinal surgery which caused external compressive force on the nerve.

**Conclusion:**

During positioning of patients into the lateral decubitus position, the course of the long thoracic nerve must be attended to carefully and the nerve should be protected from any external pressure.

## Introduction

Isolated long thoracic nerve paralysis causes weakness of the serratus anterior muscle and winging of the scapula [1,2]. The normal function of the serratus anterior muscle is to maintain the scapula in apposition to the thorax when the arm is elevated forward at the shoulder [1]. Paralysis of the serratus anterior muscle causes the scapula to rotate posteriorly on its vertical axis, producing the characteristic appearance of winging of the scapula [1,2]. So far, several traumatic and non-traumatic causes of damage to the long thoracic nerve have been reported [3,4]. We present a patient with long thoracic nerve palsy caused by the direct compression of the nerve during anterior spinal surgery. To the best of our knowledge this is the first case report of this complication in the literature.

## Case presentation

**Figure 1:**
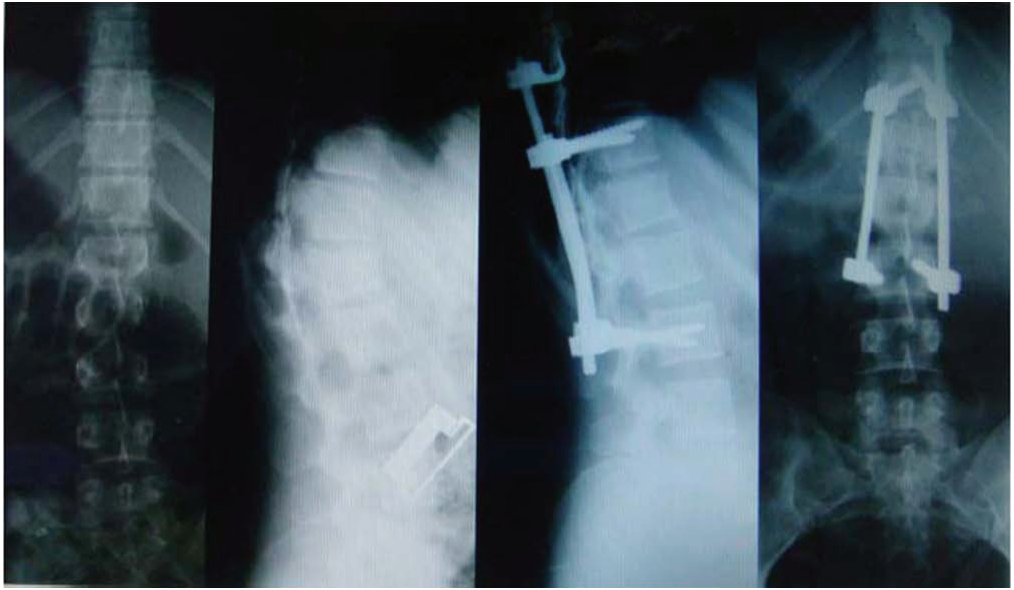
**Initial radiographs**. The initial trauma caused L1-L2 flexion-distraction injury. The patient was first treated by posterior spinal fusion and instrumentation from T11 to L3.

**Figure 2:**
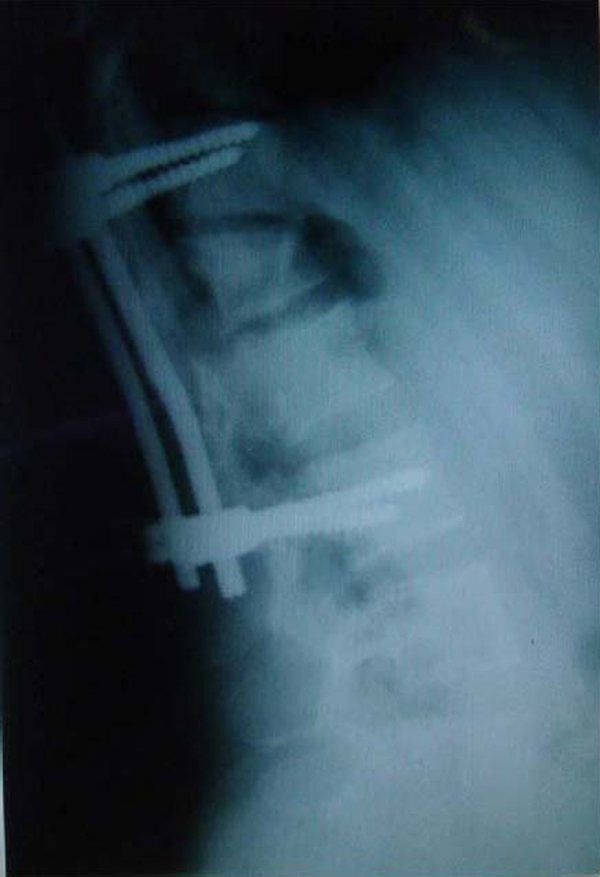
**Nine months after the primary surgery**. Nine months after the primary operation the patient presented with implant failure and thoracolumbar kyphosis.

**Figure 3:**
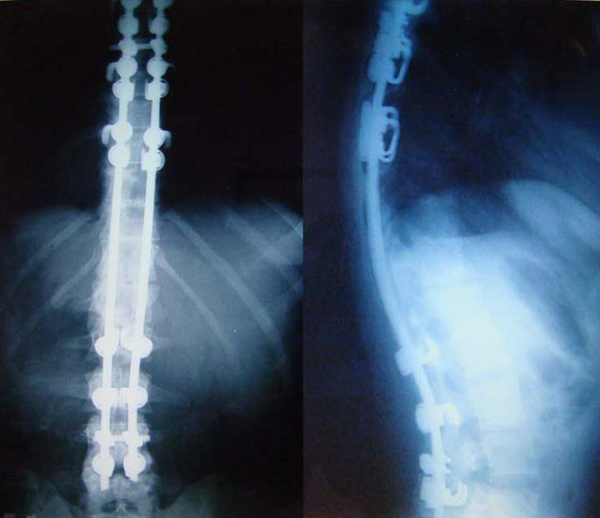
**Final radiographs**. Posteroanterior and lateral views after the three stages of surgery.

Our patient was a 23-year-old woman with a history of a car accident and an L1 flexion-distraction injury, which had been treated in another center by posterior spinal fusion and instrumentation with Cotrel-Dubousset (Figure 1). Nine months later she was referred to our department because of the failure of the instrumentation and resultant thoracolumbar kyphosis (Figure 2). She was neurologically intact. We first removed the failed instrument via a posterior approach; a week later anterior spinal release and fusion was performed. In this procedure the patient was positioned in the right lateral decubitus position (right side down) with the table flexed and a rolled towel under her axilla in order to remove the pressure from the brachial plexus and axillary vessels. Her spine was exposed by the left thoracoabdominal approach and the tenth rib resected. In the third stage,one week later, she underwent posterior spinal fusion and instrumentation. The radiographic result was satisfactory (Figure 3).

She was discharged with a total contact body brace and, after 2 weeks, on the first postoperative visit, she complained of weakness of the right upper limb during overhead activities, mild fatigue and pain in the right periscapular region. On physical examination, the right upper limb was intact for both sensory and motor innervation, but the right scapula was winged and she was not able to flex her arm forward over 60°.

**Figure 4:**
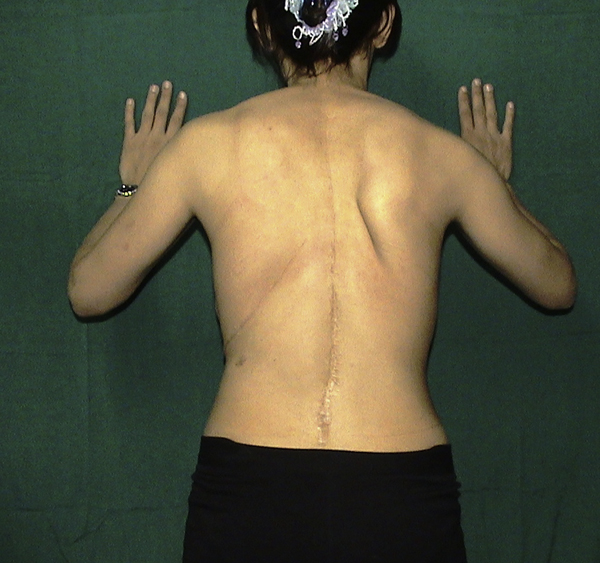
**Latest photograph**. Winging of the right scapula in pushing posture in the last follow-up visit 5 years after the surgery.

An electrodiagnostic study carried out two months later revealed an isolated long thoracic nerve lesion. The patient was treated conservatively using physical therapy and observation. As a result, the weakness of her serratus anterior muscle has lessened gradually, but on her last visit five years after the surgery some weakness was still detectable and, although she could manage her daily activities without fatigue, mild winging was still present during overhead activities (Figure 4). Finally, in the last electrodiagnostic study that was carried out 5 years after the surgery, partial and incomplete regeneration of the long thoracic nerve was reported. Although we had offered her neurolysis or nerve decompression [5]-[7] for her limited dysfunction she did not want to be operated on again.

According to the details of the electrodiagnostic studies we carried out, the site of injury was around the axillary folds. The site exactly correlated with the location in which we had inserted the axillary roll.

## Discussion

The serratus anterior is a broad muscle that has its origin in the first through to the ninth ribs. The muscle extends posteriorly and inserts on the medial border of the scapula. The long thoracic nerve, which is derived from the ventral rami of the fifth, sixth and seventh cervical nerve roots, travels over the first rib and descends on the lateral aspect of the chest wall where it divides and innervates the serratus anterior muscle [1,2].

The function of the serratus anterior muscle is to stabilize the scapula against the chest wall while elevating the arm. In initial abduction, the serratus anterior fixes the scapula so that the supraspinatus and deltoid muscles can act effectively on the humerus raising the extremity to the right angle. The serratus anterior and trapezius muscles simultaneously rotate the scapula so that the arm can be raised to the vertical position [1,2,4]. When this muscle is paralyzed, the scapula's medial border, and especially its lower angle, stands out prominently. The patient cannot raise the arm fully or push, and attempts to do so are followed by further projection or "winging of scapula" [1,2,4]. Due to its long and superficial course, the long thoracic nerve is vulnerable to damage at various levels. Several causes of its damage have been reported, including closed trauma, compression, stretching, traction, direct extrinsic force, penetrating injury, improper surgical technique, electrocution, chiropractic manipulation, various sport-related injuries including tennis, hockey, bowling, soccer, gymnastics, and weight lifting, deep massage, repetitive overhead movement, bracing for back deformity, iatrogenesis, viral infection and neuritis such as Parsonage-Turner syndrome [8]-[20]. Scapular winging can also result from repetitive or sudden external biomechanical forces that may either exert compression or place extraordinary traction in the course of the long thoracic nerve [16]-[18].

In our patient, during the anterior spinal surgery she was in the right lateral decubitus position with a rolled towel under her axilla. The operation took four hours and it appears that external compression on the nerve during surgery was the most probable cause of the nerve palsy.

**Figure 5:**
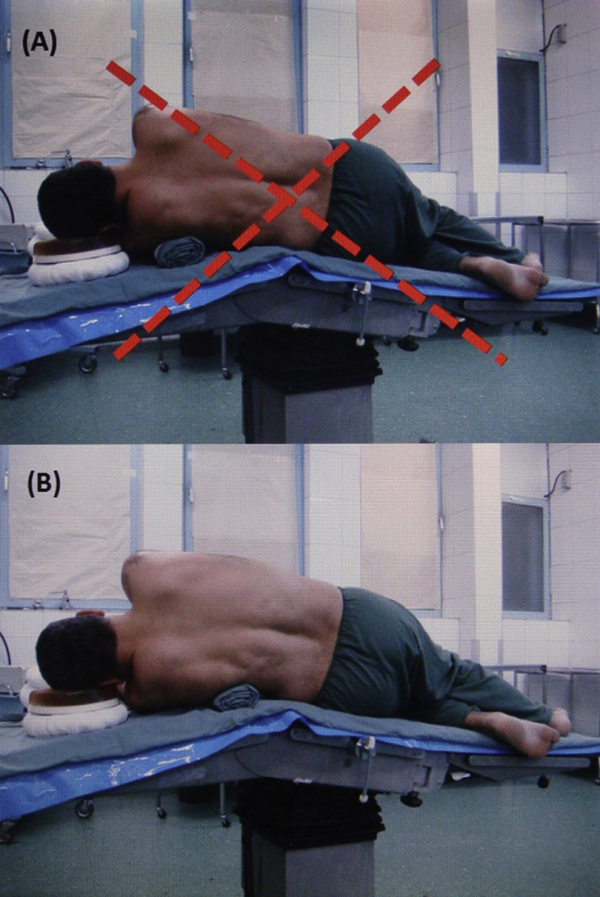
**Incorrect and correct positioning**. The image shows the previous **(A)** and current method **(B)** of our patient positioning during lateral decubitus surgery.

Since this event we have changed patient positioning during lateral decubitus surgeries. Before this, we placed the chest or axillary roll under the dependent chest wall just beneath the axilla to take pressure off the dependent shoulder and to prevent compression of the neurovascular bundle by the humeral head. Now, we place the roll about 10 cm distal to the axillary folds, roughly over the seventh to ninth ribs. This new position not only does the same as before, but also protects the main trunk of the long thoracic nerve from injury (Figure 5).

Iatrogenic injuries to the nerve have been reported during needle thoracocentesis, breast reconstruction, cardiac surgery, anesthetic block of the brachial plexus and chest tube placement [12]-[15]. In all of these iatrogenic injuries the nerve was directly traumatized, but in our patient the injury was secondary to an external compressing force.

## Conclusion

During positioning of patients into the lateral decubitus position the course of the long thoracic nerve must be attended to carefully and the nerve should be protected from any external pressure.

## Consent

Written informed consent was obtained from the patient for publication of this case report and any accompanying images. A copy of the written consent is available for review by the Editor-in-Chief of this journal.

## Competing interests

The authors declare that they have no competing interests.

## Authors' contributions

EA participated in the sequence alignment and drafted the manuscript. HB participated in the design of the study. FOK conceived of the study and participated in its design and coordination. All authors read and approved the final manuscript.
